# Loneliness and Risk for Cardiovascular Disease: Mechanisms and Future Directions

**DOI:** 10.1007/s11886-021-01495-2

**Published:** 2021-05-07

**Authors:** Elise Paul, Feifei Bu, Daisy Fancourt

**Affiliations:** Department of Behavioural Science and Health, University College London, 1-19 Torrington Place, London, WC1E 7HB UK

**Keywords:** Loneliness, Cardiovascular disease, Epidemiology, Health policy, Health promotion

## Abstract

**Purpose of review:**

In this review, we synthesise recent research on the association between loneliness and cardiovascular disease (CVD). We present evidence for mechanisms underlying this association and propose directions for future research.

**Recent findings:**

Loneliness is related to increased risk of early mortality and CVD comparable to other well-established risk factors such as obesity or smoking.

**Summary:**

Loneliness has been linked to higher rates of incident CVD, poorer CVD patient outcomes, and early mortality from CVD. Loneliness likely affects risk for these outcomes via health-related behaviours (e.g. physical inactivity and smoking), biological mechanisms (e.g. inflammation, stress reactivity), and psychological factors (e.g. depression) to indirectly damage health.

## Introduction

Accumulating evidence suggests that psychosocial risk factors such as loneliness are significant contributors to a wide range of negative health outcomes, including early all-cause mortality [1, 2], incident cardiovascular disease (CVD) [3•], and all-cause mortality in patients with CVD [4]. There are several indications that loneliness, or the subjective distress resulting from a discrepancy between desired and perceived social relationships [5], is widespread. National surveys in the UK find that 5–7% of adults feel lonely often or always [6–8]. Others find that nearly half (43%) of older adults (aged 60 and over) report feeling lonely [9]. Many of the groups more likely to be lonely are also those at an increased risk for CVD. For example, loneliness is more common amongst individuals of lower socioeconomic status (SES), poorer health, older age, and cognitive and physical impairment [10, 11]. These factors are in turn associated with higher CVD risk [12–16]. This has led to loneliness itself being identified as a public health concern [17] and developments in policy such as the establishment of a Minister for Loneliness in the UK in 2018, and the national roll-out of “social prescribing” in the NHS, the goal of which is for every general practitioner (GP) to be able to refer patients experiencing loneliness to voluntary services and community activities by the year 2023 [18].

A related but more objective construct, social isolation, has been operationalised as the number of persons with whom an individual has contact or lives, engagement in volunteering or work, and membership in social organisations or clubs [19, 20]. Although social isolation and loneliness are related and can occur together, they can also be experienced independently of one another [21]. Meta-analyses find that social isolation has comparable associations with all-cause mortality [1•] and CVD to loneliness [3•]. Whilst this review focuses specifically on loneliness rather than social isolation in relation to CVD, where studies have accounted for both, we report both sets of findings for comparison.

In this review, we provide a broad overview of current knowledge concerning whether and how loneliness influences risk for CVD incidence, prognosis, and CVD-related mortality. We begin by focusing on evidence directly linking loneliness with these outcomes. We then consider (1) psychological, (2) biological, and (3) behavioural mechanisms by which loneliness may increase the risk for CVD, emphasising findings from methodologically stronger studies when available. We conclude with a summary of the limitations of existing research as well as recommendations for future work in this area.

## Loneliness and cardiovascular disease: the evidence

Evidence linking loneliness with CVD comes from prospective studies (and meta-analyses of these studies) using population-based and CVD patient samples. In the most recent large-scale meta-analysis linking these outcomes, the pooled relative risk for incident coronary heart disease (CHD) and stroke in individuals with high versus low levels of loneliness or social isolation was 1.29 (95% confidence interval (CI) = 1.04 to 1.59) in 16 longitudinal studies [3•]. There were no significant differences in the magnitude of risk when studies examining either social isolation or loneliness were pooled separately, or in relative risk for CVD outcomes by gender, risk of confounding, or risk of measurement error.

Since this meta-analysis, other studies have repeated these findings. For example, using data from adults aged 40–69 in the large prospective population-based UK Biobank study, Hakulinen et al [22] found that in separate regression models, both loneliness and social isolation were associated with increased risk for incident acute myocardial infarction and first stroke when adjusting for demographics. Similarly, a large prospective Danish study showed independent associations of both loneliness (hazard ratio (HR) = 1.20; 95% CI = 1.03 to 1.40) and social isolation (HR = 1.23; 95% CI = 1.04 to 1.46) with CVD [23].

However, there have been mixed findings as to whether subjective feelings of loneliness or the objective construct of social isolation is more important for CVD. A 10-year follow-up study of Taiwanese adults with CVD found evidence for the role of social isolation (HR = 1.16; 95% CI = 1.07 to 1.27) but not loneliness (HR = 0.92; 95% CI = 0.80 to 1.06) with increased risk of all-cause mortality after accounting for both simultaneously and adjusting for other established risk factors [4]. Similar findings were reported using data from the Danish DenHeart study of 13,000 CVD patients, where living alone but not loneliness was associated with risk for cardiac events (myocardial infarction, stroke, ventricular tachycardia/ventricular fibrillation, and cardiac arrest) assessed from national registers in men (HR = 1.39; 95% CI = 1.05 to 1.85) at 1-year follow-up [24]. Furthermore, two other sets of analyses using data from the large population-based English Longitudinal Study of Ageing (ELSA) found that loneliness, but not indices of social isolation, was associated with increased risk for incident CVD outcomes including mortality. In the first, loneliness was associated with 30% higher risk of CVD incidence at 5-year follow-up, whilst social isolation was not [25]. A second study using the ELSA data found that loneliness but not social isolation associated with self-reported and hospital admission CVD events using objective registry data independent of sociodemographic factors [19]. So, studies remain inconclusive as to whether loneliness or social isolation are stronger predictors of CVD and CVD-related outcomes.

It is also possible that not everyone is at equal risk from social factors. For example, there have been some differential findings for men and women with respect to loneliness. Using data from the Swedish Gothenburg H70 Birth Cohort Studies, loneliness was a predictor of cardiovascular mortality in women (HR = 2.42; 95% CI = 1.04–5.65), but not in men (HR = 1.52; 95% CI = 0.78–2.96) independent of living alone [26]. Additionally, in a prospective study of population-based Finnish men (ages 42–61) who were followed for an average of 23 years, loneliness but not social isolation predicted CVD mortality (HR = 1.12; 95% CI = 1.01 to 1.24), even whilst adjusting for depressive symptoms and other confounding variables [27].

In sum, the majority of studies reviewed support subjective feelings of loneliness independent of the objective measure of social isolation as an important risk factor for incident CVD and CVD events in CVD patients. Whilst the specifics of the evidence are somewhat mixed, variation across study outcomes may depend in part on the covariates included in analyses. But there is nonetheless repeated evidence from both self-reported and objective registry data of a relationship between social factors and various CVD outcomes.

## Mechanisms linking loneliness and CVD

To date, a number of psychological, biological, and behavioural pathways to health and mortality from loneliness have been identified [28, 29]. Several researchers who have conceptualised the relationships amongst these variables begin with the observation that humans are social creatures, and that proximity to other humans is necessary for survival [30, 31]. Perceived social isolation is therefore a source of threat and danger, which activates behavioural and psychological mechanisms via biological and molecular pathways [29–31]. These mechanisms in turn lead to increased risk for premature mortality and disease [28, 32]. Identifying these pathways is crucial for developing appropriate interventions and prevention targets [28•]. Below, we review the recent evidence for these pathways whilst acknowledging that the mechanisms also likely interact with each other and operate bidirectionally.

## Psychological mechanisms

Loneliness is an established risk factor for mental ill health in meta-analyses, including depressive [33] and anxious symptoms [34], and thoughts of suicide and suicidal behaviour [11, 34, 35].

Depression in particular is hypothesised to be a mechanism linking loneliness with poor health outcomes such as CVD [28, 34]. Displays of depressive behaviour may provide a non-verbal signal to others that support and connection are needed [30]. Whilst depression and loneliness have been hypothesised to be bidirectional, recent research has specifically identified loneliness as a precursor to major depressive disorder (MDD) 2 years later, but has not found the association operating the other way [36]. However, it is likely that depression is not the sole mediator between loneliness and CVD as two recent prospective large studies found that the relationship between loneliness and CVD mortality persisted even whilst adjusting for depressive symptoms [19, 27].

Another psychological mechanism that could explain the link between loneliness and CVD is stress. Stress is a key aspect of the experience and definition of loneliness [5]. Humans are inherently social animals and perceived isolation may activate a stress response designed to facilitate survival in the short-term by seeking proximity to other humans, but also by producing hypervigilance to threats to compensate for the lack of mutual protection [30, 31]. Psychological stress has been shown to result from loneliness, along with biological stress responses (see “biological mechanisms” below), and stress-related health behaviours (see “behavioural mechanisms”) [37, 38]. Increased stress can in turn lead to compromised physiological resilience [39]. Excessive worries over time that are not attenuated by the removal of stressors can also become more generalised anxiety, which has been shown to be bidirectionally related to loneliness [36].

Additionally, research has also examined other negative behaviours including anger and hostility in relation to loneliness, finding that these behaviours are heightened in lonely individuals, perhaps in part because increased vigilance for social threats in the absence of perceived social connectedness may protect lonely individuals in the short term [30]. However, these negative states, along with stress and depression, can also act as a short-term precipitant of major cardiac events such as acute coronary syndrome [40, 41]. Therefore, evidence suggests that negative moods, stress, and poor mental health are all pathways connecting loneliness to cardiovascular events.

## Biological mechanisms: neuroendocrine, immune, cardiometabolic, physiological, and epigenetic

Evidence for biological pathways through which loneliness impacts CVD comes from both human and animal studies [30, 32]. Mortality risk from loneliness remains elevated even when controlling for psychological and behavioural factors, suggesting a role for biological mechanisms [28•]. Underlying many of these biological pathways are psychological mechanisms discussed above including depression and chronic elevated stress [28, 29], This is likely because hormonal, vascular, and immune responses are set off by the danger to one’s survival from perceived social isolation and loneliness [28, 32, 40, 42]. These biological responses in turn can, when chronic and sustained, lead to adverse health consequences which place the individual at risk for premature mortality and disease development. Some of these key biological pathways are outlined below.

### Neuroendocrine pathways

The stress associated with loneliness has led to much research focused on atypical (exaggerated or blunted) neuroendocrine responses to stress as a major pathway through which subjective feelings of loneliness influence CVD risk [28, 32, 42, 43]. Loneliness activates the body’s central stress response system—the hypothalamic pituitary adrenal (HPA) axis—in order to prepare the body to deal with potential threats that may occur as a result of perceived social isolation [32, 42]. The HPA axis sets into motion a “flight or fight” response by secreting the stress hormone cortisol, which typically shows higher levels upon morning awakening and a blunted circadian rhythm across the day [42•]. Over time, chronic stress can lead to chronically elevated cortisol levels, which are associated with CVD incidence and poorer prognosis, as well as CVD risk factors such as diabetes and systolic blood pressure [44].

The majority of the available evidence supports an exaggerated HPA response in association with loneliness; larger increases in cortisol upon awakening, higher concentrations of circulating cortisol, and diminished sensitivity of glucocorticoid receptors have all been found in lonely individuals [32•]. Specifically in reaction to stressful events, momentary, day-to-day sampling studies have found higher levels of cortisol reactivity in lonely compared to non-lonely individuals [45, 46]. However, there is still some inconsistency in the literature: a systematic review of studies measuring neuroendocrine responses to acute laboratory-induced stressors such as giving a public speech found that although most studies reviewed reported exaggerated stress responses in lonelier individuals, one study found blunted cortisol production in lonelier women but not men, whilst two others reported no difference in cortisol levels between lonely and non-lonely individuals [43].

Responses have also not been as clearly found for other hormones. Studies examining loneliness and neuropeptides have found no association with catecholamines (e.g. epinephrine, norepinephrine, dopamine) secreted in response to signals from the sympathetic nervous system during acutely stressful events [47, 48]. Therefore, further research on the role of stress hormones in mediating the relationship between loneliness and CVD using larger longitudinal studies is needed.

### Immune function

A second possible biological mechanism linking loneliness with CVD is weakened immunity through increased inflammation [28, 31, 32]. Cytokines (inflammatory proteins within the immune system) not only coordinate the body’s inflammatory response, but also initiate so-called “sickness behaviours”, which include fatigue and social withdrawal and are a part of individuals’ response to illness [31, 49]. The resulting inflammation may enable sick individuals to prepare for the possibility of infection or harm [31, 50]. Therefore, proinflammatory response that occurs in loneliness may be seen as a protection in the short term against threats to safety, as humans who are disconnected from others are more vulnerable to illness [31]. However, this inflammation may also increase CVD risk when sustained.

In terms of specific cytokines and other inflammatory markers associated with loneliness, a systematic review of the evidence linking loneliness and inflammatory reactions to acute laboratory-induced stress (e.g. a performance task or pharmacological induction with amphetamines) found loneliness to be associated with increased proinflammatory cytokines and glycoproteins such as IL-6, tumour necrosis factor alpha (TNFa), interleukin-1 beta (IL-1B), monocyte chemoattractant protein 1 (MCP-1), and fibrinogen [43]. However, a more recent meta-analysis focusing on observational studies of loneliness, social isolation, and inflammation found higher levels of interleukin-6 (IL-6) but not fibrinogen in association with loneliness [51]. This study also found no relationship with the inflammatory marker C-reactive protein (CRP) [51]. Even though CRP is a biomarker associated with depression (which we have already discussed as one psychological pathway linking loneliness with CVD) [52], the relationship between CRP and loneliness has also not been shown by other studies, including a study that found no evidence that CRP acts as a mediator between loneliness and depression [53], and another study finding that loneliness was associated with higher inflammatory markers such as insulin-like growth factor 1 (IGF-1) but not CRP or fibrinogen, even though social isolation was related to these factors [54]. So, it appears that only some inflammatory markers are related to loneliness.

Additionally, in their recent review, Li and Xia [32] argued that another biological mechanism underlying the association between psychosocial stress due to loneliness and CVD is oxidative stress. Longitudinal research on this topic is needed given evidence for a bi-directional relationship between loneliness and immune response [55]. However, it highlights the breadth of immune markers now being considered in relation to loneliness.

### Cardiometabolic response

A third possible pathway through which loneliness increases the risk for CVD is cardiometabolic changes such as elevated blood pressure [28, 43] and heart rate variability [43]. In a recent meta-analysis, loneliness was related to elevated diastolic blood pressure, increased vascular resistance, and lower heart rate variability in response to acute laboratory-based stressors [43]. A more recent narrative review reported lonely individuals to have greater total peripheral vascular resistance and increased risk of hypertension [32]. However, findings from the ELSA epidemiological study (not included in the Brown et al. [43] review) showed that social isolation but not loneliness was associated with increases in both systolic and diastolic blood pressure [20]. Since these reviews, one study of healthy young women (18–29 years) found that those with greater levels of chronic loneliness had lower resting HRV and significantly greater increases in HRV after exposure to laboratory-induced state of loneliness [56]. Women who were chronically lonely also showed blunted HRV reactivity to a cognitive challenge task [56].

Psychosocial stress associated with loneliness may also influence CVD via changes in gut microbiota. Activation of the HPA axis initiates an immune response that in turn decreases microbial diversity, and leads to further HPA axis activation [57]. These stress-induced changes in gut microbiota have been shown to increase risk for CVD in clinical associations in human studies and experimental studies in animals [58, 59]. However, research has yet to determine whether the stress associated with loneliness in particular induces changes in gut microbiota which are in turn associated with CVD.

In considering other biological pathways, small genetic contributions to loneliness have been reported [60] and chronic social isolation in mice and rats has been shown to lead to epigenetic changes in the brain [61, 62]. However, research on whether loneliness induces epigenetic changes in humans is needed, particularly changes in the epigenome that are associated with CVD risk.

## Behavioural mechanisms

Finally, the quality of one’s social relationships may have an indirect effect on CVD by promoting healthy behaviours such as exercise, healthy eating, and not smoking or abusing alcohol [63]. Feeling lonely on the other hand may impair the capacity to self-regulate and avoid negative behaviours such as excessive alcohol use which reduce stress and tension in the short-term, but are ultimately harmful for health in the long-term [30]. Evidence supporting these behavioural mechanisms comes from studies showing loneliness is associated with a lack of physical activity [20, 64], smoking [20, 65, 66], poor diet [65], and problematic alcohol use [67–69].

In other studies, the association between loneliness and CVD has been found to be attenuated when adjusting for behavioural factors that could lie on the causal pathway and thereby act as pathways to CVD. In models that adjusted for potential mechanisms such as physical inactivity, smoking, and body mass index, the relationship between loneliness and mortality has been shown to be attenuated in participants who had earlier acute myocardial infarction [22]. However, one recent longitudinal study of older adults (ELSA) showed that the impact of loneliness on CVD persisted, even when accounting for physical inactivity, smoking, and alcohol use [19], suggesting that these behavioural factors may only partially explain the relationship with CVD-related outcomes and also that more research is needed to establish the direction of these associations. Further, whether it is loneliness or social isolation that has the strongest association with health behaviours is still being questioned as another study using data from the ELSA cohort found that social isolation but not loneliness at baseline was associated with a number of health risk behaviours (e.g. decreased likelihood of regular physical activity, lower fruit and vegetable intake, smoking) [70]. Lonely individuals who smoked were, however, less likely to have stopped smoking at follow-up than non-lonely individuals [70].

In sum, alongside psychological and biological pathways connecting loneliness to CVD risks, there are also a number of potential behavioural pathways that likely interact with other pathways. For example, sedentary behaviours are themselves associated with higher depression and inflammatory response [71, 72]. This highlights both the range of mechanisms connecting loneliness to CVD but also the interconnectedness of these mechanisms.

## Conclusions and implications

Research demonstrates that loneliness is an independent and modifiable risk factor for CVD. The available evidence implicates psychological factors such as stress, depression, atypical physiological reactivity, and neuroendocrine responses to stress, increased inflammation, and harmful behaviours such as smoking, drinking alcohol, and poor diet as possible mechanisms.

Evidence of effective interventions for loneliness is limited [9]. A review of interventions to reduce social isolation and loneliness in older adults found that whilst most did report effectiveness, the quality of evidence was weak [73]. Although participants in social-prescribing interventions (e.g. group exercise, arts classes) report satisfaction with the programmes and reduced feelings of loneliness [74, 75], there have to date not been studies examining mortality or CVD outcomes of these non-medical interventions (social prescribing) [28•]. Therefore, in addition to deeper exploration of the magnitude of the relationship between loneliness and CVD risk amongst different populations (including groups at low and high risk of developing CVD) and the mechanisms underlying this relationship (in particular how combinations of mechanisms can act synergistically with one another), future research is also encouraged to investigate interventions that could help to reduce the negative health effects of loneliness. Such research could play an important role in the design of holistic care strategies for people at risk of or living with CVD and help to reduce the burden of managing CVD for health and social care services.
